# 234 Optimized Swab-Based Skin Screening Improves Detection of Carbapenem-Resistant Acinetobacter baumannii

**DOI:** 10.1017/ash.2026.10610

**Published:** 2026-06-23

**Authors:** Jenna Rasmusson, Jean Barth, Debra Apenhorst, Sarah Bellows Mahler, Leah Siple, Alyssa Olson, Michelle Meyer, Rebecca Faller, Priya Sampathkumar

**Affiliations:** 1 Mayo Clinic; 2 Mayo Clinic Rochester; 3 Mayo Clinic, Rochester, MN

## Abstract

**Background:** Carbapenemase-producing organisms (CPO) are a serious public health threat. The Centers for Disease Control and Prevention (CDC) guidelines for combating CPO includes a recommendation to screen selected high risk patients. We describe a program to identify and screen patients at risk for CPO. Setting: An academic, tertiary care center with 2,059 licensed beds and 74,359 admissions a year. **Methods:** A report was created in the electronic medical record (EMR) to identify adult patients admitted in the previous 24 hours who were from countries and states with known CPO transmission based on address and zip code. A nursing protocol was subsequently developed in the EMR to facilitate CPO screening for eligible patients at admission. After the CPO order is placed, an electronic communication is sent via the EMR alerting the patient care team of the order, the rationale for screening, details on swab collection, and links to a toolkit with resources to help answer patient questions. A single perirectal swab is obtained by the patient’s primary nurse and is tested for Klebsiella pneumoniae Carbapenemase (KPC), New Delhi metallo-β-lactamase (NDM), oxacillinase-48 (OXA-48), and Verona integron-encoded metallo-β-lactamase (VIM) by polymerase chain reaction (PCR). Patients may refuse testing. **Results:** From May 2018 through November 2025, 6,039 patients were identified for CPO screening using case-finding report. Of these patients, 2,424 had CPO swabs completed. Eighteen patients with CPO were identified with an overall yield of 0.75% on admission screening. There was one patient who tested positive for KPC, 10 patients who tested positive for NDM, and 5 patients who tested positive for OXA-48. Two patients were identified to have both NDM and OXA-48. **Conclusions:** The EMR can be leveraged for early identification and screening of patients with epidemiologically significant pathogens. Use of the nursing protocol has enabled IPAC to complete timely CPO surveillance and prevent transmission to other patients. Protocols within the EMR can be effectively replicated for a timely response to emerging infections.

